# Supplementary figures and images for: Diet-induced variability of the resistin gene (Retn) transcript level and methylation profile in rats
Source: BMC Genet. 2015 Sep 17;16:113. doi: 10.1186/s12863-015-0270-4 (PMC4574077; doi:10.1186/s12863-015-0270-4)

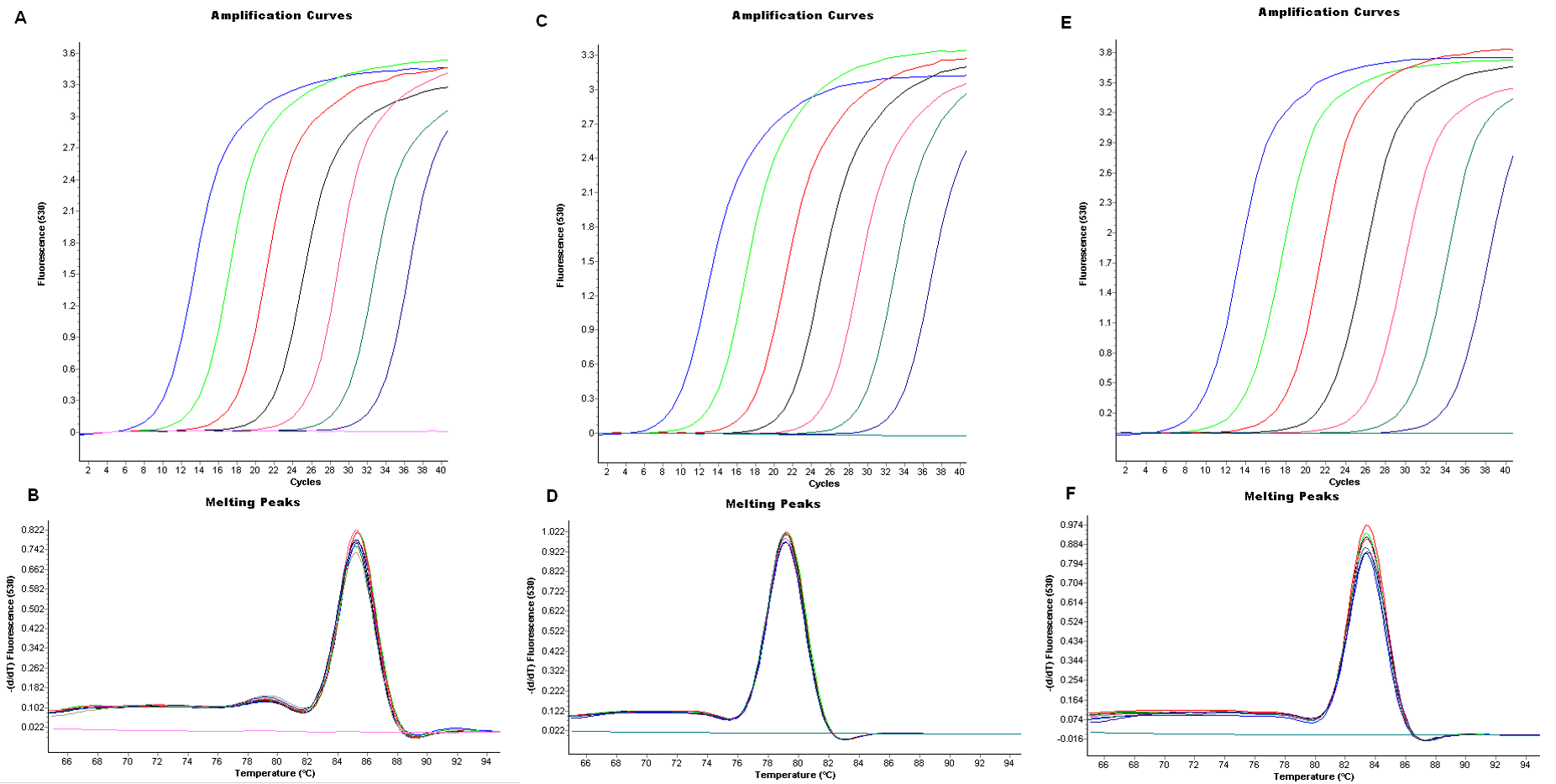

Supplement: Additional file 2: Figure S1. — Representative qPCR amplification curves of serial dilutions and melting curves of the Retn gene (A and B) as well as two reference genes: Hprt (C and D) and Tbp (E and F). (BMP 5071 kb) [file 12863_2015_270_MOESM2_ESM.bmp]
